# Inflammation and altered metabolism impede efficacy of functional electrical stimulation in critically ill patients

**DOI:** 10.1186/s13054-023-04664-7

**Published:** 2023-11-06

**Authors:** T. S. O. Jameson, M. K. Caldow, F. Stephens, L. Denehy, G. S. Lynch, R. Koopman, A. Krajcova, T. Urban, S. Berney, F. Duska, Z. Puthucheary

**Affiliations:** 1 Nutritional Physiology Group, Department of Sport and Health Sciences, College of Life and Environmental Sciences, University of Exeter, Exeter, Devon UK; 2 Centre for Muscle Research, Department of Anatomy and Physiology, The University of Melbourne, Melbourne, VIC Australia; 3 Department of Physiotherapy, School of Health Sciences, The University of Melbourne, Melbourne, Australia; 4 Department of Anaesthesia and Intensive Care Medicine, Third Faculty of Medicine, Charles University, FNKV University Hospital, Srobarova 50, 10034 Prague, Czech Republic; 5 Department of Physiotherapy Division of Allied, Health Austin Health, Austin, TX USA; 6 William Harvey Research Institute, Barts and The London School of Medicine, Queen Mary University of London, London, UK; 7 Adult Critical Care Unit, Royal London Hospital Barts Health NHS Trust, London, UK

**Keywords:** Muscle wasting, Critical illness, Exercise, Rehabilitation, Gene expression

## Abstract

**Background:**

Critically ill patients suffer from acute muscle wasting, which is associated with significant physical functional impairment. We describe data from nested muscle biopsy studies from two trials of functional electrical stimulation (FES) that did not shown improvements in physical function.

**Methods:**

*Primary cohort*: single-centre randomized controlled trial. Additional healthy volunteer data from patients undergoing elective hip arthroplasty. *Validation cohort*: Four-centre randomized controlled trial. *Intervention*: FES cycling for 60-90min/day. *Analyses*: Skeletal muscle mRNA expression of 223 genes underwent hierarchal clustering for targeted analysis and validation.

**Results:**

Positively enriched pathways between healthy volunteers and ICU participants were “stress response”, “response to stimuli” and “protein metabolism”, in keeping with published data. Positively enriched pathways between admission and day 7 ICU participants were “FOXO-mediated transcription” (admission = 0.48 ± 0.94, day 7 = − 0.47 ± 1.04 mean log_2_ fold change; *P* = 0.042), “Fatty acid metabolism” (admission = 0.50 ± 0.67, day 7 = 0.07 ± 1.65 mean log_2_ fold change; *P* = 0.042) and “Interleukin-1 processing” (admission = 0.88 ± 0.50, day 7 = 0.97 ± 0.76 mean log_2_ fold change; *P* = 0.054). Muscle mRNA expression of UCP3 (*P* = 0.030) and DGKD (*P* = 0.040) decreased in both cohorts with no between group differences. Changes in IL-18 were not observed in the validation cohort (*P* = 0.268). Targeted analyses related to intramuscular mitochondrial substrate oxidation, fatty acid oxidation and intramuscular inflammation showed PPARγ-C1α; (*P* < 0.001), SLC25A20 (*P* = 0.017) and UCP3 (*P* < 0.001) decreased between admission and day 7 in both arms. LPIN-1 (*P* < 0.001) and SPT1 (*P* = 0.044) decreased between admission and day 7. IL-18 (*P* = 0.011) and TNFRSF12A (*P* = 0.009) increased in both arms between admission and day 7. IL-1β (*P* = 0.007), its receptor IL-1R1 (*P* = 0.005) and IL-6R (*P* = 0.001) decreased in both arms between admission and day 7. No between group differences were seen in any of these (all p > 0.05).

**Conclusions:**

Intramuscular inflammation and altered substrate utilization are persistent in skeletal muscle during first week of critical illness and are not improved by the application of Functional Electrical Stimulation-assisted exercise. Future trials of exercise to prevent muscle wasting and physical impairment are unlikely to be successful unless these processes are addressed by other means than exercise alone.

**Supplementary Information:**

The online version contains supplementary material available at 10.1186/s13054-023-04664-7.

## Background

Critically ill patients suffer from acute muscle wasting, resulting in significant physical functional impairment [1, 2]. After discharge, patients struggle to regain muscle mass, strength and functional capacity [3]. This results in substantial functional limitations that persist in 70% of patients at 6–12 months, and 30% remain carer-dependent [2]. At 12 months, only 56% of previously employed patients return to work, with physical disability and fatigue as associative factors [4]. The scale and characteristics of this muscle wasting have now been described: rates of 2–3% loss per day are seen consistently, with preferential loss of Type II muscle fibres and evidence of non-excitable muscle membrane [1, 5]. Loss of muscle mass is the result of altered protein homeostasis from depressed muscle protein synthesis and increased protein degradation [1, 6].

Two parallel storylines have appeared in the literature in regard to acute muscle wasting and subsequent physical impairment: firstly, that neither physical nor nutritional interventions have been successful at preventing or treating this physical impairment [7, 8] and secondly, that the underpinning biology of altered protein homeostasis described in observational studies increasingly converges on common physiological and metabolic alterations [9].

Resistance exercise is a major stimulant of muscle protein synthesis, and exercise rehabilitation has grade I level evidence for improving physical function in other populations [10]. However, exercise interventions have not proven to be efficacious in the critically ill population. A systematic review of 43 trials demonstrated that physical rehabilitation in isolation cannot prevent or restore the loss of function associated with critical illness [11]. A narrative review of nine trials of increased substrate delivery did not demonstrate improvements in physical function [12]. Recently, the TARGET trial demonstrated no difference in quality of life or return to work rates with increased calorie delivery [13]. Increasing nutrition delivery in the EPANIC trial resulted in decreased muscle quality [14].

Persistent inflammation in critically ill patients is well described and contributes to both short- and long-term morbidity [15]. Intramuscular inflammation and cellular infiltration were first described in 1991 [16]. Tumour necrosis alpha (TNFα), hypoxia-inducible factor-1 (HIF1) and interleukin-6 (IL-6) and interleukin-10 (IL-10) have all been reported to increase [17–19]. The cellular infiltrates have been characterized to be CD68-positive macrophages, though not seen in all studies [17, 20, 21]. Mitochondrial dysfunction was first described in 2002 and confirmed in subsequent studies [22–24]. Inflammation and hypoxia signalling lead to altered carbohydrate and lipid substrate utilization, and insulin resistance [18]. The combination of these processes results in a range of alterations of cellular bioenergetics [18, 24]. Muscle protein synthesis, the facilitative mechanism of maintaining and increasing muscle mass in humans, is highly energy dependent [25]. Unless the bioenergetic impairment and intramuscular inflammation are addressed, it would seem unlikely that the usual stimuli of muscle protein synthesis (resistance exercise and amino acids) will be successful and therefore address the physical impairment of critical illness survivors.

In this study, we describe data from nested muscle biopsy studies within two trials of functional electrical stimulation (FES) that did not shown improvements in physical function [26, 27]. We hypothesized that the application of FES in the very early stages of critical illness may not alleviate the inflammation and alteration of cellular bioenergetics, which would explain the lack of efficacy of the intervention. If so, functional electrical stimulation may be an appropriate co-intervention to trial alongside interventions to address the underpinning biology.

## Methods

### Primary cohort

Single-centre prospective randomized controlled trial. Inclusion criteria were: mechanical ventilation and predicted ICU length of stay ≥ 7 days. FES cycling was performed 90min/day for 7 days/week. Full details including ethical approval are available in the study protocol and the original publication [26, 28].

### Validation cohort

Nested sub-study of a four-centre (Australian and USA) randomized controlled trial. Inclusion criteria were: mechanical ventilation and sepsis, severe sepsis or systemic inflammatory response syndrome and ICU stay ≥ 4 days post-randomization. FES cycling was performed 60min/day, ≥ 5 days/week unilaterally with the unstimulated leg acting as a control. Full details including ethical approval are available in the original publication [27].

### Healthy volunteers

In addition to the critically ill participants, metabolically healthy participants undergoing elective hip arthroplasty in spinal or epidural anaesthesia were recruited. After obtaining a prospective written informed consent, vastus lateralis muscle sample was taken by open technique as soon as the muscle was exposed during surgery.

### Functional electrical stimulation

Supine bikes with FES module RT300 System (© Restorative Therapies, Baltimore USA) Inc. 2005–2016. LB100108 Version 37 were used in both cohorts. Electrical stimulation was provided to the following muscles: rectus femoris; hamstrings; and gluteals and gastrocnemius using large size (20cm) electrodes. Electrodes were placed on both legs although muscle stimulation was only provided to the leg randomized to receive FES. The cycling only leg received sham electrical stimulation. The intervention was provided in addition to usual care rehabilitation by registered physiotherapists, experienced in critical care rehabilitation, who were not blinded to the randomization group.

#### Primary cohort

The intervention had a goal of being delivered 90 min per day, seven days a week. After warm-up phase (5 min of passive cycling), participants received therapy consisting of functional electrical stimulation or active cycling with duration adjusted per protocol and participants’ tolerance followed by relaxation phase (5 min of passive cycling). FES impulses had a pulse width of 250 μs, pulse frequency of 40 Hz and the lowest output per channel (in a range 0–60 mA) that allowed locomotive movement of lower extremities. To increase the intervention workload, both resistance (3–10 Nm) and cycling cadence were increased incrementally. When appropriate, FES was supplemented with protocolized goal-directed mobilization. Standard rehabilitation group received the usual care (SC), which was monitored but not protocolized. It consisted of passive muscle rehabilitation, in-bed exercises and standard mobilization, as appropriate.

#### Validation cohort

The intervention had a goal of being delivered for at least five out of seven day per week. Stimulation was set at 20–30 mA for all stimulated muscles and was titrated to achieve muscle contraction and participant comfort. The intervention was delivered to a single leg. Pulse width (microseconds) was set to 250 for average sized legs and was increased to 300 if the leg was oedematous. Frequency (Hertz) was set at a mean of 43.5 and increased as appropriate to 50 to induce a strong muscle contraction.

### Sample collection

In all patients, using an aseptic technique under local anaesthetic injected into skin and fascia (i.e. 2 ml of 1% lignocaine) muscle biopsies were obtained from the vastus lateralis muscle 10 cm above the patella. Biopsies were performed by ICU medical staff using the percutaneous needle technique originally described by Bergstrom [29]. Biopsies were frozen in liquid nitrogen and stored at -80°C until biochemical analysis was performed.

### Laboratory analyses: primary cohort and healthy volunteers

Total RNA was extracted from ∼20 mg frozen muscle tissue using TRI Reagent® (Thermo Fisher Scientific, MA, USA) according to the manufacturer’s protocol. Total RNA was quantified spectrophotometrically at 260 nm (NanoDrop ND-2000 Spectrophotometer; Thermo Fisher Scientific, MA, USA), and RNA purity was determined as the ratio of readings at 260/280 nm. Reverse transcription of RNA was carried out using a commercially available kit (SuperScript™ VILO™ cDNA Synthesis Kit, Thermo Fischer Scientific, MA, USA). Muscle mRNA expression of 224 genes from pathways associated with inflammation, insulin signalling, myogenesis, protein turnover, stress response, substrate metabolism, extracellular matrix remodelling, cellular amino acid transport and associated nuclear transcription factors were determined using custom designed RT-PCR OpenArray™ plates (Thermo Fischer Scientific, MA, USA) in combination with a QuantStudio 12K Flex Real-Time PCR system (Thermo Fischer Scientific, MA, USA) as we have previously described [30, 31]. A total of 223 genes were included in the final analysis. Relative muscle gene expression was calculated using the 2^−ΔΔCT^ method. Gene expression values are presented as the log_2_ fold change in mRNA abundance calculated relative to healthy volunteers with the median cohort age, and the geometric mean of housekeeping genes β-actin and β2 microglobulin.

### Laboratory analyses-validation cohort

Total RNA was extracted from 10 TO 20 mg muscle tissue using a commercially available kit, according to the manufacturer’s instructions (RNeasy Fibrous Tissue Mini Kit, Qiagen, VIC, Australia). RNA quality and concentration were determined using the NanoDrop 2000 spectrophotometer (Thermo Scientific, Waltham, MA, USA). RNA was transcribed into cDNA using iScript cDNA Synthesis Kit (Bio-Rad Laboratories, NSW, Australia). qPCR was performed using the Bio-Rad CFX384 PCR system (Bio- Rad Laboratories), in triplicate with reaction volumes of 10 ul, containing SsoAdvanced™ Universal SYBR® Green Supermix (Bio-Rad Laboratories), forward and reverse primers and cDNA template. The efficacy of *B2M* (beta2-microglobulin) as an endogenous control was examined using the equation 2^−ΔCq^. *B2M* mRNA was not influenced by the intervention and therefore was deemed an appropriate endogenous control. Data were analysed using a comparative quantification cycle (Cq) method where the amount of target relative to B2M is given by 2^−ΔΔCq^. Primers were designed using NCBI Primer BLAST from gene sequences obtained from GenBank and have bene described elsewhere [32] or are listed in Additional file 1: Table S1.

### Data analysis

#### Untargeted functional enrichment analysis

Comparing the group difference in every gene would result in a considerable loss in statistical power due to the correction required for 223 individual statistical tests (to avoid family-wise error), leading to missing meaningful effects, or reporting type I errors. To overcome this, we used all 223 genes in the pathway approach to determine what pathways were perturbed by critical illness per se (corrected for family-wise error prior to pathway analysis). After applying a false discovery rate (FDR) of < 5% to adjust for family-wise error on these gene lists, in line with our previous work [30, 33] functional enrichment analysis was performed against the Reactome database using the PANTHER overrepresentation test (binomial test with no correction; released 2022–02-02; PANTHER version 17.0). The original gene list of 213 genes, as opposed to the whole genome, was used as the background. Network diagrams were generated using ShinyGo (version 0.76.2) using the Reactome pathway database and the whole genome as the background.

#### Targeted analysis and validation

The pathway analysis demonstrated key alterations in inflammatory and substrate utilization pathways. Forty genes specifically associated with substrate metabolism and inflammation were then selected a priori for a targeted investigation, by a researcher (ZP) not involved in data analysis. This gene list underwent an initial screening to identify genes which were differently expressed in ICU participants at admission compared with control participants using separate unpaired t tests, yielding 27 genes (substrate metabolism = 17, inflammation = 15). The effect of FES *versus* standard care on muscle gene expression was investigated using two-way repeated measure mixed effect models with time (admission *vs* day 7) and group (control *vs* FES) as factors. Furthermore, the expression of these 27 a priori selected genes underwent hierarchical clustering using Multiple Experiment Viewer (MeV, version 4.9.0) to generate a cluster of genes for validation in muscle samples obtained from the second FES study. The effect of FES *versus* standard care on muscle gene expression in the second FES study was investigated using two-way repeated measure mixed effect models with time (admission *vs* discharge) and leg condition (standard care *vs* FES) as factors.

## Results

Out of 150 participants enrolled into the primary study, samples were taken from 31 participants (16 intervention, 15 controls) to form primary study cohort at admission and day 7 (Additional file 1: Figure S1). In the secondary cohort, samples were taken from 11 participants (5 interventions, 6 controls) at admission and ICU discharge (Additional file 1: Figure S2). The characteristics of these participants are shown in Table 1. A total of 17 participants undergoing elective hip replacement surgery were recruited as healthy volunteers.

**Table 1:** Baseline study subject characteristics

	Primary cohort (Czech Republic)		Validation cohort (Australia)		Czech Republic
	Intervention n = 16	Control n = 15	Intervention n = 5	Control n = 6	Healthy Volunteers
Age	58 ± 17	64 ± 11	65 ± 8.5	60 ± 17.8	64 ± 14
Sex (M/F)	12/4	10/5	3/2	3/3	9/8
BMI	29.2 ± 5.9	33.3 ± 8.1	26 ± 3.5	22 ± 2.9	29.9 ± 3.4
APACHE II	22 ± 5	27 ± 7	21 ± 6.3	17 ± 4.3	N/A
ICU LOS pre-recruitment	1.4 ± 0.8	1.4 ± 0.7	2 ± 0.9	4 ± 2.2	N/A
History of diabetes (%)	6/10 (38%)	7/8 (47%)	0/0 (0%)	2/6 (33%)	0/17 (0%)
Pre-admission CCI	2.9 ± 2.0	3.7 ± 2.8	2 ± 1.4	3 ± 2.9	N/A
Diagnostic category					
Trauma	8 (50%)	4(25%)	0 (0%)	0 (0%)	
Surgical	3 (19%)	1 (7%)	2 (40%)	1 (16%)	
Medical	5 (31%)	10 (68%)	3 (60%)	5 (84%)	
Sepsis	5 (31%)	5 (33%)	5 (100%)	6 (100%)	

Data are mean ± SD unless stated. *BMI* Body Mass Index, *APACHE II* Acute Physiology and Chronic Health Evaluation, *ICU LOS* Intensive Care Unit Length of Stay, *CCI* Charlson Comorbidity Index

### Untargeted gene expression: pathway analysis

After applying a FDR < 5% correction, 137 genes were differentially expressed between healthy volunteers and ICU participants at ICU admission. The three most positively enriched pathways associated with this gene list were “Cellular response to stress” (healthy volunteers = -0.20 ± 0.50, ICU participants = 0.93 ± 0.74 mean log_2_ fold change from a nominal healthy volunteer; *P* = 0.047), “Cellular response to stimuli” (healthy volunteers = -0.20 ± 0.50, ICU participants = 0.93 ± 0.74 mean log_2_ fold change;* P* = 0.062) and “Metabolism of proteins” (healthy volunteers = − 0.12 ± 0.51, ICU participants = 0.93 ± 0.76 mean log_2_ fold change; *P* = 0.089) (Additional file 1: Figs. 1A, 2A, and Additional file 1: Table S2 for gene lists).

**Fig. 1:**
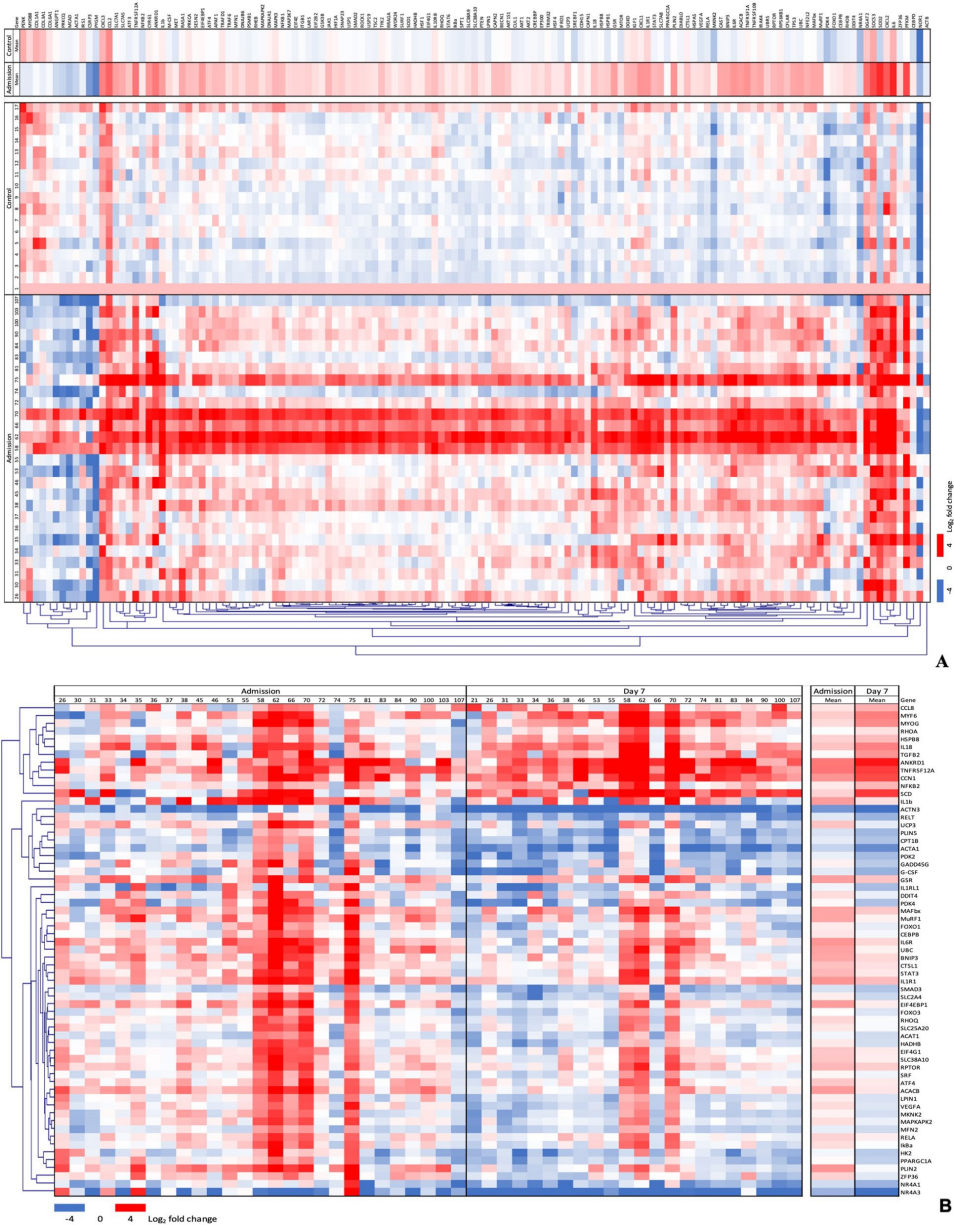
Heat maps of intramuscular log_2_ fold change differential gene expression between critically ill patients on day 1 (admission) versus healthy volunteers undergoing elective hip surgery (**A**) and critically ill patients on day 7 (**B**)

**Fig. 2:**
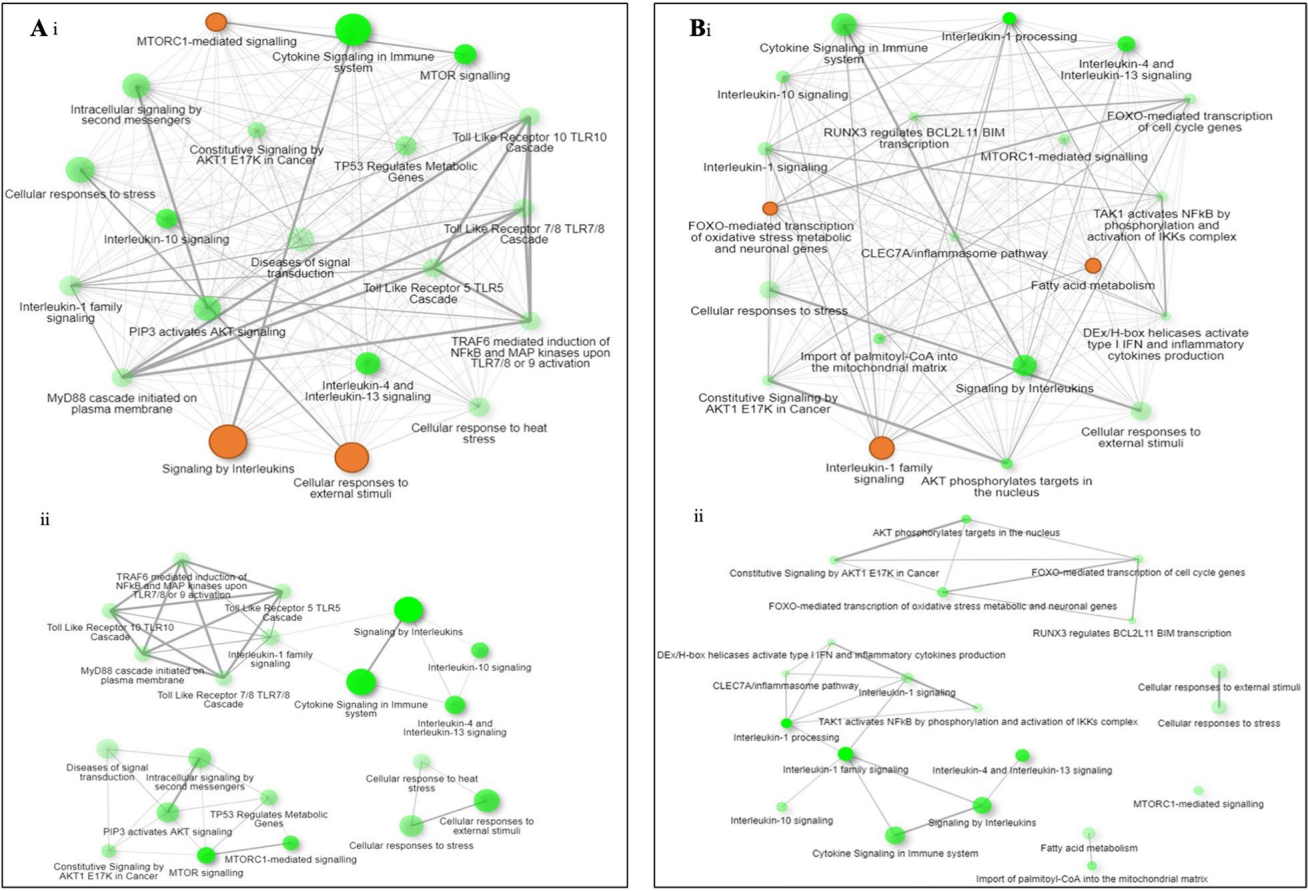
Network maps of muscle gene expression generated from the Reactome database using overrepresentation tests. Top maps (i) provide an overview of all enriched pathways, whereas bottom maps (ii) provide detail on the top three most enriched pathways shown in orange. **A** Differentially enriched pathways between critically ill patients on day 1 (admission) versus healthy volunteers undergoing elective hip surgery. **B** Differentially enriched pathways between critically ill patients on day 1 (admission) versus day 7

After applying a FDR < 5% correction, 63 genes were differentially expressed between ICU participants at admission compared with the same participants at day 7. The three most positively enriched pathways associated with this gene list were “FOXO-mediated transcription of oxidative stress, metabolic and neuronal genes” (admission = 0.48 ± 0.94, day 7 = − 0.47 ± 1.04 mean log_2_ fold change; *P* = 0.042), “Fatty acid metabolism” (admission = 0.50 ± 0.67, day 7 = 0.07 ± 1.65 mean log_2_ fold change; *P* = 0.042) and “Interleukin-1 processing” (admission = 0.88 ± 0.50, day 7 = 0.97 ± 0.76 mean log_2_ fold change; *P* = 0.054) (Figs. 1B and 2B). The small numerical difference between means at some time points is a result of pathways containing both genes that are up (positive expression value)- and downregulated (negative expression value).

### Targeted gene expression (Mitochondrial substrate oxidation)

**Fig. 3:**
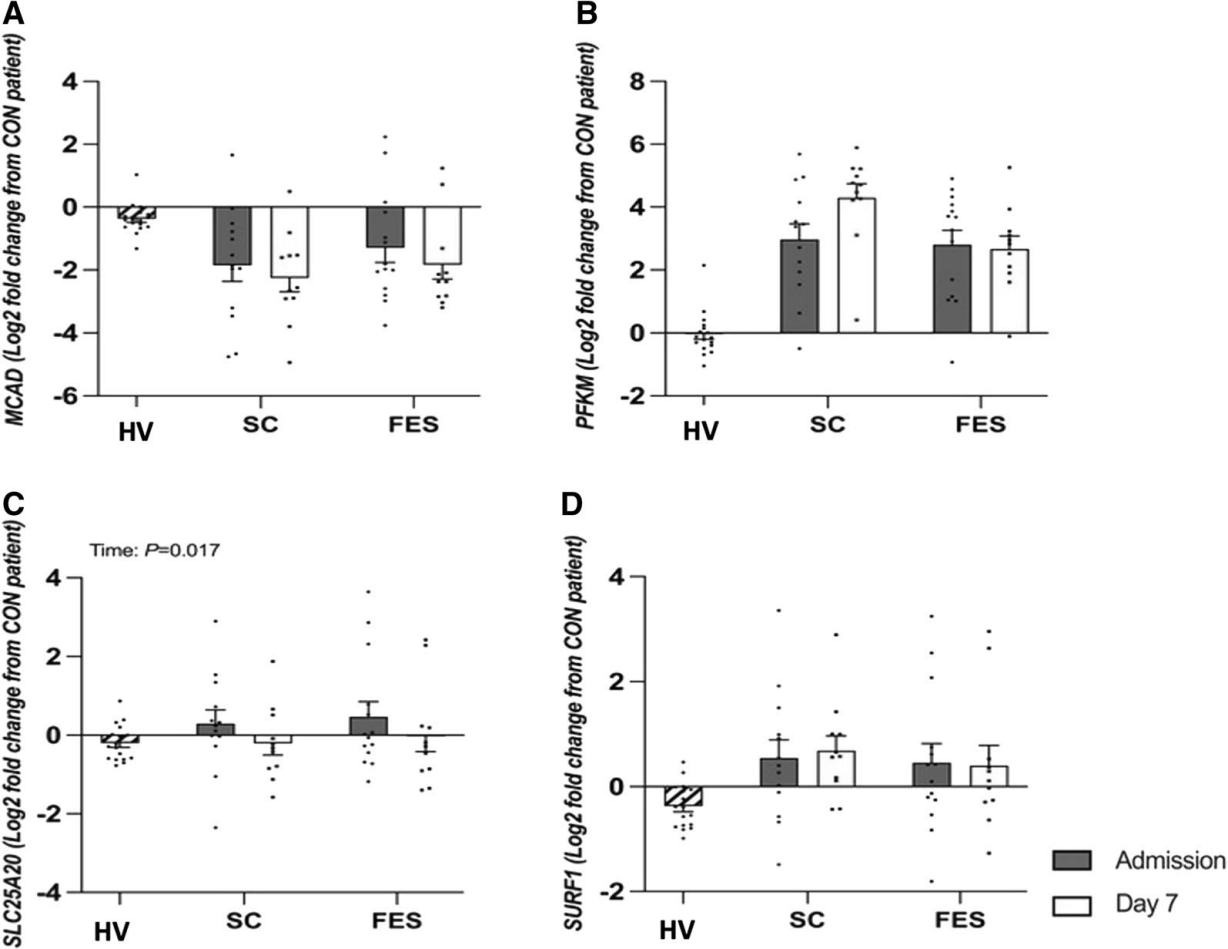
Targeted fold change differences in skeletal muscle gene expression regulating mitochondrial substrate oxidation between healthy volunteers (HV) and critically ill patients either receiving routine standard of care (SC) or Functional Electrical Stimulation (FES) of genes; *MCAD* Acyl-CoA Dehydrogenase Medium Chain; *PFKM* Phosphofructokinase; *SLC25a20* Solute Carrier Family 25 Member 20; and *SURF-1* Surfeit locus protein 1. Data are expressed as mean ± SEM

**Fig. 4:**
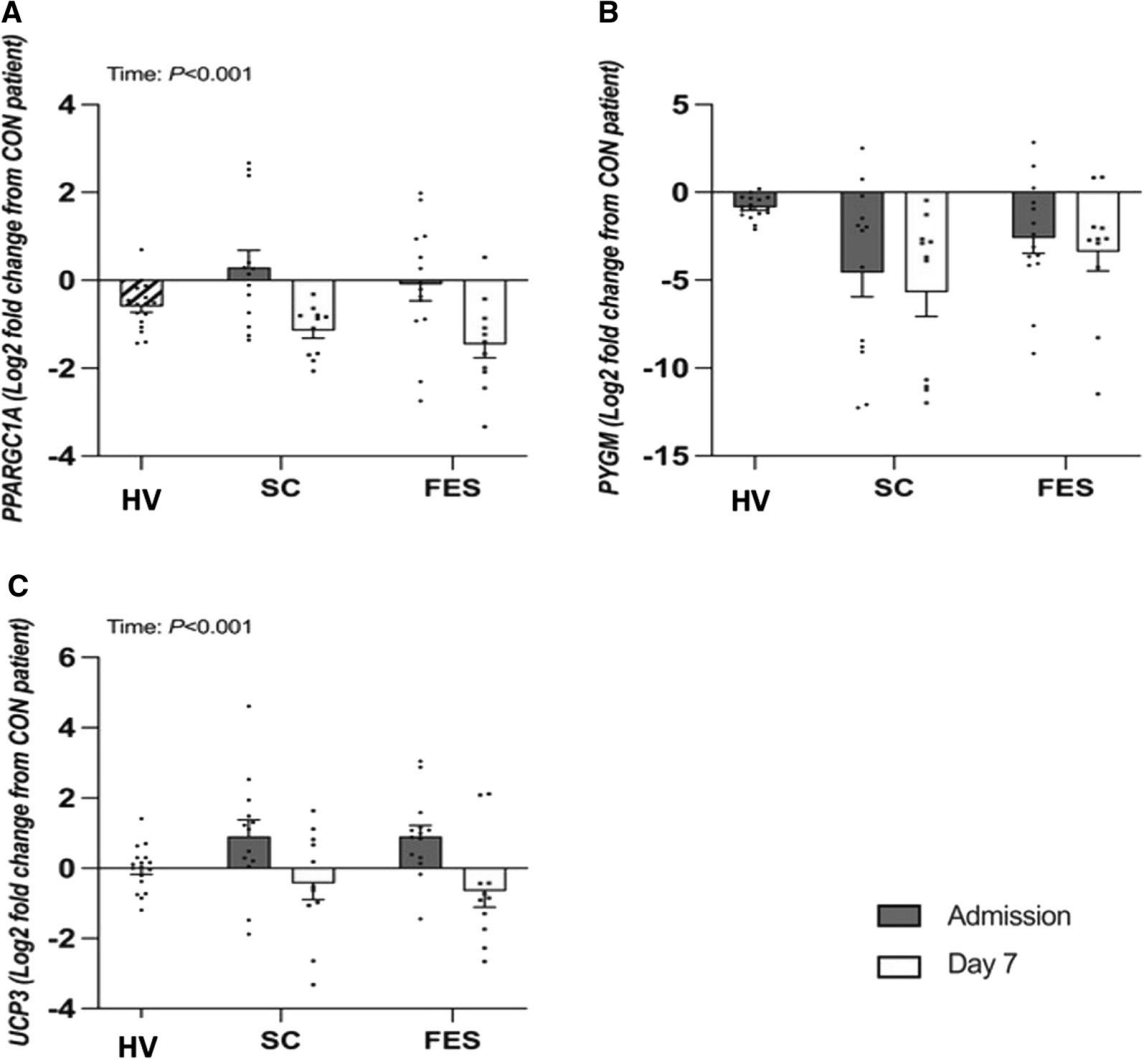
Targeted fold change differences in skeletal muscle gene expression regulating mitochondrial substrate oxidation between healthy volunteers (HV) and critically ill patients either receiving routine standard of care (SC) or Functional Electrical Stimulation (FES) of genes; PPARGC1A = Peroxisome proliferator-activated receptor gamma coactivator 1-alpha; PYGM = Glycogen phosphorylase; UCP3 = Uncoupling Protein 3. Data are expressed as mean ± SEM

The log_2_ fold change in muscle expression of PPARγ-C1α; (*P* < 0.001), SLC25A20 (*P* = 0.017) and UCP3 (*P* < 0.001) decreased between admission and day 7 in both control and FES arms with no group difference (all interaction effect; *P* > 0.05, Figs. 3 and 4, Additional file 1: Table S3). No differences were seen between admission and day 7 in MCAD, PFKM, SURF-1 and PYGM gene expression (all p > 0.05).

### Targeted gene expression (Lipid synthesis)

**Fig. 5:**
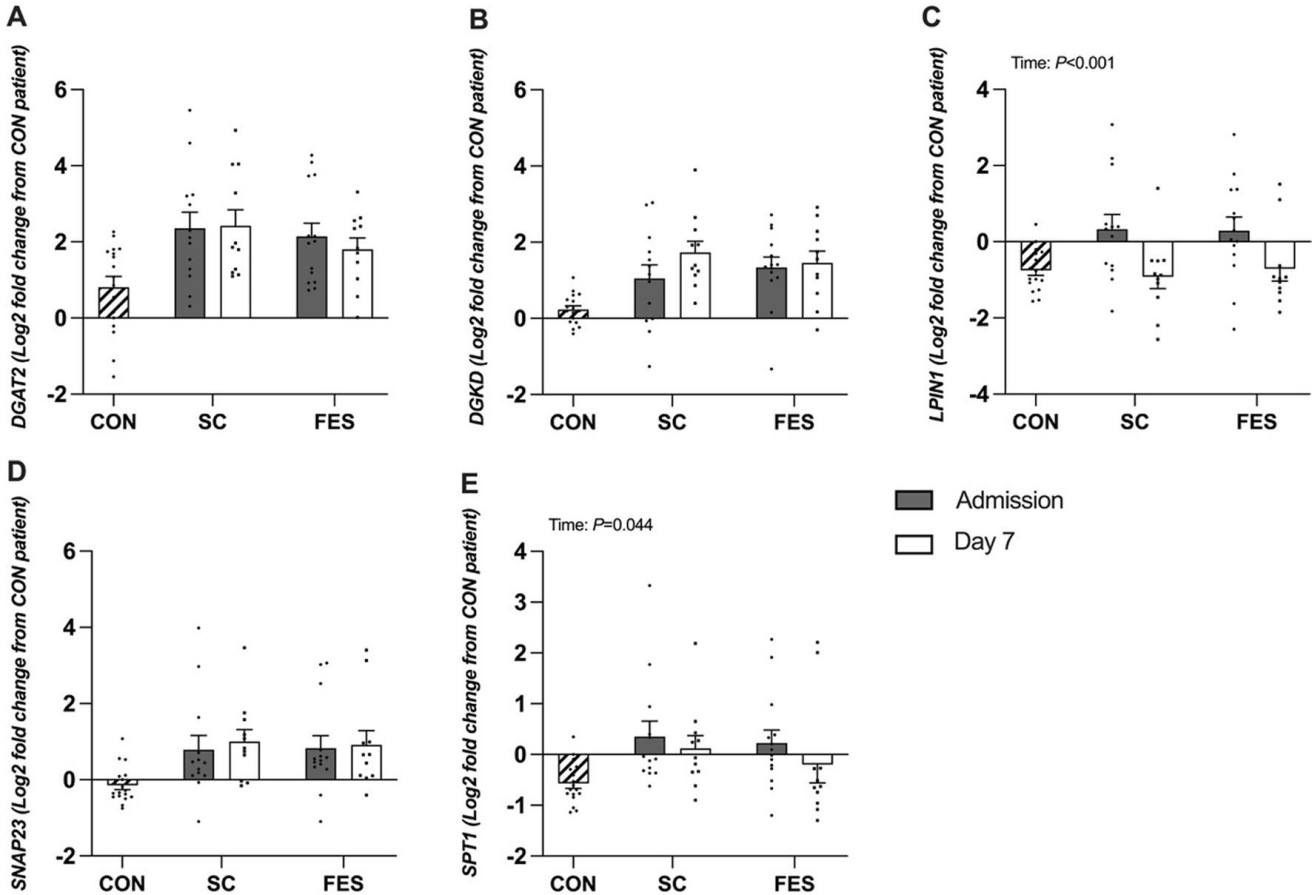
Targeted fold change differences in skeletal muscle genes regulating lipid synthesis between healthy volunteers (HV) and critically ill patients either receiving routine standard of care (SC) or Functional Electrical Stimulation (FES). DGAT2 = Diacylglycerol O-Acyltransferase 2; DGKD = Diacylglycerol Kinase Delta; LPN1 = Lipin-1; SNAP23 = Synaptosome Associated Protein 23; and SPT1 = Serine palmitoyltransferase 1. Data are expressed as mean ± SEM

The log_2_fold change in muscle expression of LPIN-1 (*P* < 0.001) and SPT1 (*P* = 0.044) decreased between admission and day 7 in both control and FES arms with no group differences (both interactions effects; *P* > 0.05, Fig. 5, Additional file 1: Table S3). No differences were seen between admission and day 7 in DGKD, DGAT2 and SNAP 23 gene expression (all p > 0.05).

### Targeted gene expression (Intramuscular Inflammation)

**Fig. 6:**
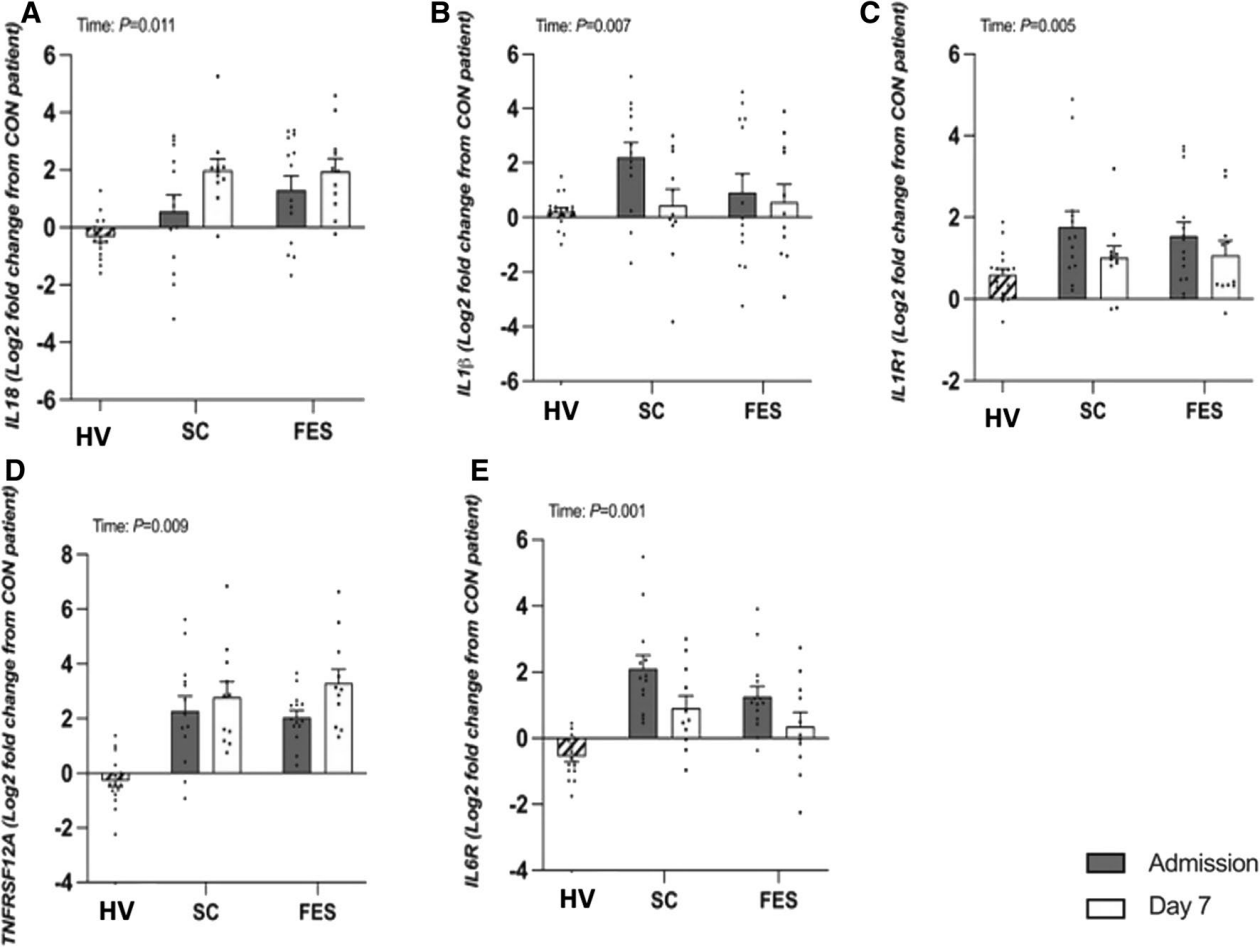
Targeted fold change differences in skeletal muscle genes regulating intramuscular inflammation between healthy volunteers (HV) and critically ill patients either receiving routine standard of care (SC) or Functional Electrical Stimulation (FES). IL-1b = Interleukin 1B; IL-1R1 = Interleukin 1 receptor 1; IL-18 = Interleukin 18; Il6R = Interleukin 6 receptor; and TNFRSF12A = Tumour necrosis factor receptor superfamily member 12A; Data are expressed as mean ± SEM

The log_2_fold change in muscle mRNA expression of Il-18 (*P* = 0.011) and TNFRSF12A (*P* = 0.009) increased in control and FES arms between admission and day 7, with no group differences (both interaction effects; *P* > 0.05). The mRNA expression of Il-1β (*P* = 0.007), its receptor Il-1R1 (*P* = 0.005) and Il-6R (*P* = 0.001) decreased in control and FES arms between admission and day 7 with no group differences (all interaction effects; *P* > 0.05, Fig. 6, Additional file 1: Table S3). No differences were seen in other inflammatory gene expression, (Additional file 1: Figure S3).

### Validation of gene expression

**Fig. 7:**
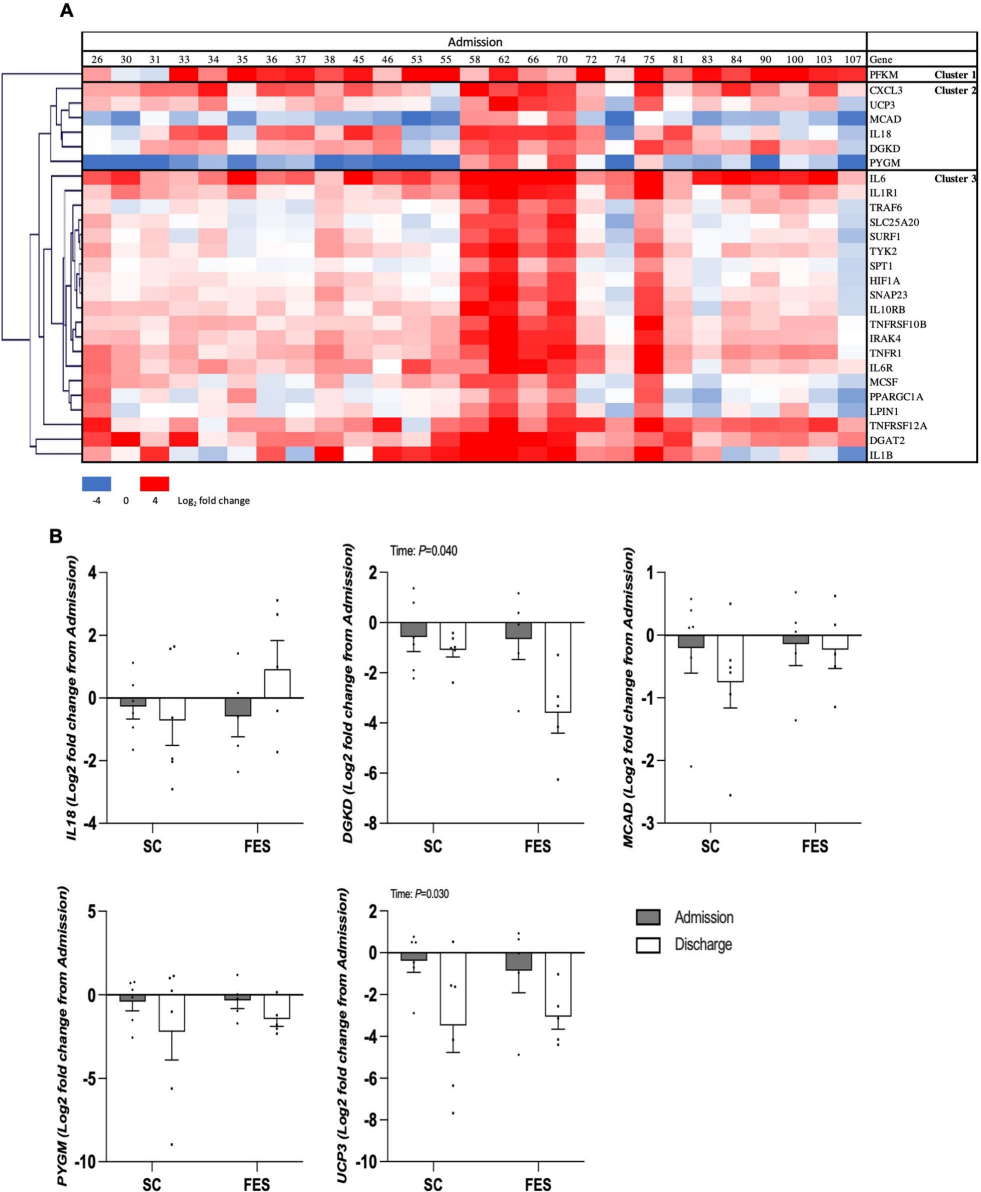
**A** Hierarchal clustering on 27 a priori selected genes which were differentially expressed between healthy volunteers (HV) and ICU patients at admission, identifying cluster for validation. **B** Differential gene expression in the validation cohort between ICU admission and discharge in ICU patients undergoing unilateral Functional Electrical Stimulation (FES) with the contralateral leg receiving standard care (SC). IL-18 = Interleukin-18; DGKD = Diacylglycerol Kinase Delta; MCAD = Acyl-CoA Dehydrogenase Medium Chain; PYGM = Glycogen phosphorylase; and UCP3 = Uncoupling Protein 3. Data are presented as fold change with gene expression normalized to patient’s admission values for each group, mean ± SEM

Hierarchal clustering was performed on 27 a priori selected genes which were differentially expressed between healthy volunteers and ICU participants at admission to identify a cluster in an unbiased manner that could be validated in a separate cohort (Fig. 7A). Clustering identified 3 distinct clusters and “cluster 2” was selected as it contained genes associated with both inflammation and substrate utilization, and however, CXCL3 could not be measured in validation samples due to methodological issue.

In a similar fashion to patterns of mRNA expression observed in the primary cohort, muscle mRNA expression of UCP3 (P = 0.030) decreased in both arms between admission and day 7 in the validation cohort with no group differences (interaction effect; P = 0.672). Further, a decrease in DGKD mRNA expression (*P* = 0.040) was observed in the validation cohort with no group differences (interaction effect; *P* = 0.128). Changes in IL-18 (intramuscular inflammation) were not observed in the validation cohort (*P* = 0.268, Fig. 7B, Additional file 1: Table S4).

## Discussion

We performed an analysis of muscle biopsy samples collected prospectively as nested physiological sub-studies from two randomized controlled trials of Functional Electrical Stimulation (FES) in critically ill patients. The primary aim was to understand the underpinning mechanism of the failure of the intervention to improve patients’ functional outcomes. Gene expression data demonstrated an increase in intramuscular inflammation and altered substrate utilization, which was seen in both trials (primary and validation cohorts). Pathway analyses demonstrate these alterations to be consistent along congruent biological processes, deepening our understanding of these phenomena.

These data are consistent with previous data on the metabolic phenotype of skeletal muscle in critical illness, and importantly, we were not able to detect any significant or consistent changes of this pattern by the application of FES. The presence of critical illness seems to strongly dominate the possible influence of FES on muscle biology. These findings offer an insight into the mechanism of the lack of efficacy of exercise interventions to improve functional outcomes in critically ill patients.

### Altered substrate utilization impedes both muscle mass and function

Decreases in pyruvate dehydrogenase kinase gene expression reflect the effects of hypoxia and inflammation on carbohydrate metabolism preventing pyruvate from entering the Krebs cycle, and diverting to lactate production (i.e. the Pasteur effect [34]). Decreases in lipid metabolism are seen at various points of the fatty acid oxidization process, with decrease in carnitine-acylcarnitine translocase and Acetyl-CoA acetyltransferase-1 gene expression seen. Decreases in Lipin-1 and increases in Stearoyl-CoA gene expression may be the causal pathways for accumulation of intramyocellular lipid seen in critically ill patients [20]. These metabolic derangements lead to the bioenergetic failure seen in critical illness, which will impede both muscle protein synthesis and muscle contractile force [18, 35]. These derangements would need to be addressed if FES protocols are to be effective in maintaining muscle mass and improving muscle function in critically ill patients.

### Intramuscular inflammation may be the driver of anabolic resistance

Increases in pro-inflammatory cytokine gene expression within the skeletal muscle of critically ill patients have been well described, though there is significant heterogeneity in patients affected and concentrations seen, which is reflected in this analysis. The pathway analyses suggest that inflammation precedes metabolic alterations in substrate utilization. These metabolic alterations contribute to muscle dysfunction and generate an environment not conducive to favourable adaptations with normal anabolic signalling, i.e. anabolic resistance [36]. Anabolic resistance is prevalent in critical care settings decreasing nutritional protein incorporation into skeletal muscles [37]. These data add to the growing body of evidence that, in the same vein as exercise interventions, nutritional interventions are unlikely to be successful in the presence of intramuscular inflammation. Importantly, these data offer a caveat to the lack of efficacy of the FES intervention: there may still be a role for FES in patients without inflammation, or once inflammation has resolved.

### Implications for future trials and clinical practice

FES represents the current state of the art in delivering resistance exercise (as a stimulus for muscle protein synthesis) to those unable to volitionally participate. These data represent a call for a cessation for trials of resistance exercise as standalone interventions, and a rationale to couple resistance exercise with co-interventions to address altered substrate utilization and intramuscular inflammation. The role of early resistance exercise in critically ill patients remains unclear, though a clear distinction must be made with early mobilization, for which there is sufficient data to show that this improves outcomes. The benefits of early mobilization have its causal roots across many processes (e.g. related to sedation-holds, delirium management, patient engagement, lung aeration) that are unrelated to maintenance of skeletal muscle mass and function [38]. The presence of altered substrate utilization and inflammation is likely to be causal in regard to muscle wasting, and future research may be better applied to exercise interventions once these processes have resolved.

### Implications for future mechanistic research

The relationship between intramuscular inflammation, altered substrate utilization and loss of muscle mass has been described [18]. A similar relationship with muscle function has been less so. The lack of efficacy of FES interventions in critically ill patients could be attributed to compromised muscle adaptive responses and altered contractile activity. Exploring these potential mechanisms was outside the scope of this study, though in future such studies might identify what must be addressed to enhance therapeutic efficacy. These mechanisms are likely those that might similarly interfere with the expected adaptations to exercise, nutrition and/or pharmacological interventions in older adults or cancer cachexia where inflammation is a common mechanism [39, 40]. Loss of neuromuscular junction (NMJ) integrity has been reported in settings of acute and chronically elevated inflammation, such as in cancer cachexia and sarcopenia [41]. The temporary (or long-term) loss of a functioning connection between pre-synaptic and post-synaptic structures at the NMJ could account for some of the muscle wasting and weakness in critical care settings and compromise the efficacy of FES protocols. The dystrophin–glycoprotein complex (DGC) has well-described roles in normal force transmission, especially in the context of muscle diseases such as Duchenne muscular dystrophy, with evidence accumulating for other roles in anabolic and vascular signalling [42].

### Strengths and limitations

This analysis has several strengths, based around the a priori decision to embed muscle biology analysis in clinical trials, the blinding of the laboratory analyses from clinical data, and the use of two independent cohorts from two groups in different continents using the same intervention. Limitations include the small number of samples in the validation cohort, and the limited amount of tissue available for analysis, requiring focused validation of biological processes and the minor differences in trial protocols between cohorts. Nevertheless, these data represent, to our knowledge, both the first embedded muscle biology analyses within a critical care exercise or rehabilitation trial, and the first paper to use primary and validation cohorts to demonstrate findings within skeletal muscle of critically ill patients.

## Conclusions

Intramuscular inflammation and altered substrate utilization are persistent in skeletal muscle during first week of critical illness and are not improved by the application of Functional Electrical Stimulation-assisted exercise. Future trials of exercise to prevent muscle wasting and physical impairment are unlikely to be successful unless these processes are addressed by other means than exercise alone.

### Supplementary Information

**Additional file 1.** Online Supplement.

## Data Availability

Source data can be obtained from the corresponding author upon reasonable request.

## Declarations

### Competing of interests

Z. A. P. reports personal fees from Faraday Pharmaceuticals, Lyric Pharmaceuticals, Bioage Fresenius Kabi, Nestlé, Orion and GlaxoSmithKline, outside the submitted work.
