# Supplementary material for: Inflammation and altered metabolism impede efficacy of functional electrical stimulation in critically ill patients
Source: Crit Care. 2023 Nov 6;27:428. doi: 10.1186/s13054-023-04664-7 (PMC10629203; doi:10.1186/s13054-023-04664-7)
Supplement: Supplementary file 1 — Additional file 1. Online Supplement. [file 13054_2023_4664_MOESM1_ESM.docx]

**ONLINE SUPPLEMENT**

Figure S1: CONSORT flowchart of recruitment for the primary cohort


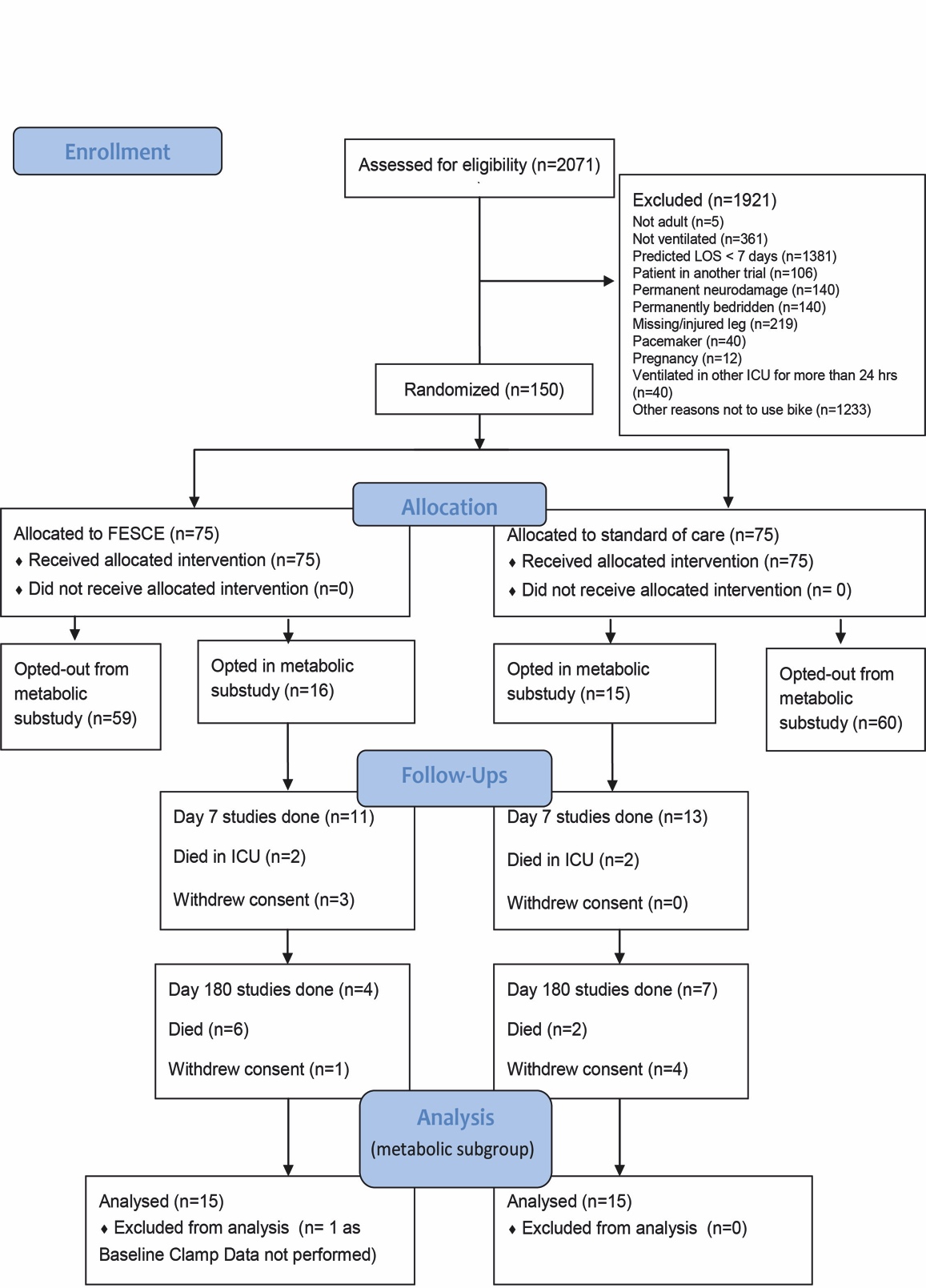


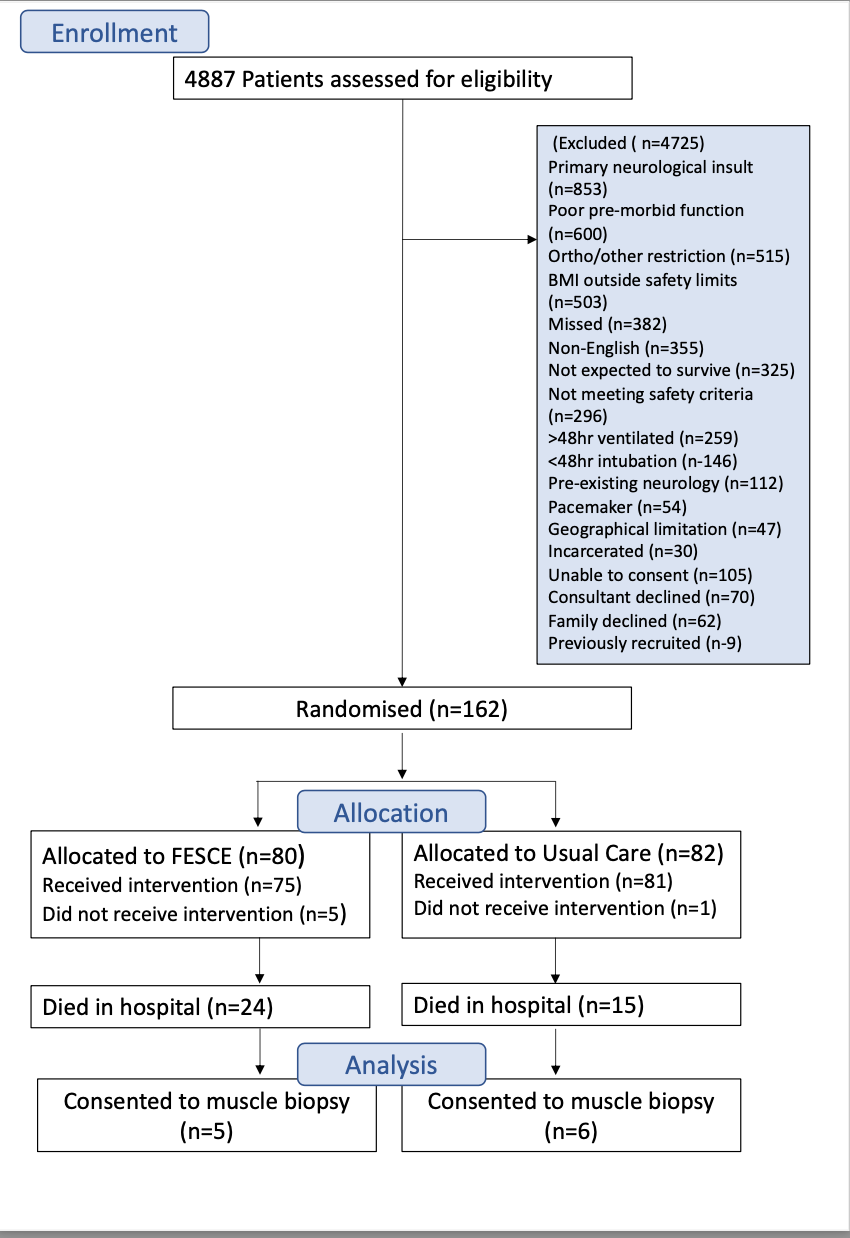


Figure S2: CONSORT flowchart of recruitment for the validation cohort

Table S1: Gene primers for validation cohort analyses

| **Gene** | **Accesssion Number** | **Forward (5’-3’)** | **Reverse (5’-3’)** | **Amplicon length** |
| --- | --- | --- | --- | --- |
| *PYGM* | NM_005609.4 | TCACACTCGTAAAGGACCGC | TCTGTAGCGTCCGTCCCATA | 180 |
| *ACADM* | NM_000016.6 | GGCCGTGACCCGTGTATTAT | CTGCAGCATCGCCCGAA | 77 |
| *IL18* | NM_001386420.1 | GCTGAAGATGATGAAAACCTGGA | GAGGCCGATTTCCTTGGTCA | 115 |
| *UCP3* | NM_003356.4 | AGCCCCCTCGACTGTATGAT | ACTTTCATCAGGGCCCGTTT | 149 |
| *DGKD* | NM_152879.3 | TCAGATCCGACAGAAGACCATC | TGACTTTGCCGTTTTGGCAT | 130 |

| **Cellular response to stress (*P*=0.0513)** | **Metabolism of proteins (*P*=0.0615)** | **Cellular responses to external stimuli (*P=0.0683)*** | **mTORC-1 mediated signalling (P0.0913).** |
| --- | --- | --- | --- |
| Ras-related GTP-binding protein A RRAGA | Eukaryotic translation initiation factor 4 gamma 1 EIF4G1 | Ras-related GTP-binding protein A RRAGA | Eukaryotic translation initiation factor 4 gamma 1 EIF4G1 |
| Proline-rich AKT1 substrate 1 AKT1S1 | Leucine--tRNA ligase, cytoplasmic LARS1 | Proline-rich AKT1 substrate 1 AKT1S1 | Ras-related GTP-binding protein A RRAGA |
| DnaJ homolog subfamily A member 1 DNAJA1 | C-C motif chemokine 2 CCL2 | DnaJ homolog subfamily A member 1 DNAJA1 | Proline-rich AKT1 substrate 1 AKT1S1 |
| Polyubiquitin-C UBC | Ras-related GTP-binding protein A RRAGA | Polyubiquitin-C UBC | Eukaryotic translation initiation factor 4E EIF4E |
| Cyclic AMP-dependent transcription factor ATF-4 ATF4 | Macrophage colony-stimulating factor 1 CSF1 | Cyclic AMP-dependent transcription factor ATF-4 ATF4 | Serine/threonine-protein kinase mTOR MTOR |
| Endoplasmic reticulum chaperone BiP HSPA5 | Polyubiquitin-C UBC | Endoplasmic reticulum chaperone BiP HSPA5 | Ribosomal protein S6 kinase beta-1 RPS6KB1 |
| Cyclic AMP-dependent transcription factor ATF-3 ATF3 | F-box only protein 32 FBXO32 | Cyclic AMP-dependent transcription factor ATF-3 ATF3 | Regulatory-associated protein of mTOR RPTOR |
| DnaJ homolog subfamily B member 6 DNAJB6 | Cyclic AMP-dependent transcription factor ATF-4 ATF4 | DnaJ homolog subfamily B member 6 DNAJB6 | GTP-binding protein Rheb RHEB |
| Sestrin-2 SESN2 | Endoplasmic reticulum chaperone BiP HSPA5 | Sestrin-2 SESN2 | Sodium-coupled neutral amino acid transporter 9 SLC38A9 |
| Vascular endothelial growth factor A VEGFA | Cyclic AMP-dependent transcription factor ATF-3 ATF3 | Vascular endothelial growth factor A VEGFA | Eukaryotic translation initiation factor 4E-binding protein 1 EIF4EBP1 |
| Heat shock factor protein 1 HSF1 | Translation initiation factor eIF-2B subunit beta EIF2B2 | Heat shock factor protein 1 HSF1 |  |
| Serine/threonine-protein kinase mTOR MTOR | Actin, cytoplasmic 1 ACTB | Serine/threonine-protein kinase mTOR MTOR |  |
| Glycogen synthase kinase-3 beta GSK3B | Eukaryotic translation initiation factor 4E EIF4E | Glycogen synthase kinase-3 beta GSK3B |  |
| Transcription factor p65 RELA | Ubiquitin carboxyl-terminal hydrolase 19 USP19 | Transcription factor p65 RELA |  |
| Cellular tumor antigen p53 TP53 | Nuclear factor NF-kappa-B p100 subunit NFKB2 | Cellular tumor antigen p53 TP53 |  |
| Hypoxia-inducible factor 1-alpha HIF1A | CCN family member 1 CCN1 | Hypoxia-inducible factor 1-alpha HIF1A |  |
| Superoxide dismutase [Mn], mitochondrial SOD2 | Transcription factor p65 RELA | Superoxide dismutase [Mn], mitochondrial SOD2 |  |
| Regulatory-associated protein of mTOR RPTOR | Cellular tumor antigen p53 TP53 | Regulatory-associated protein of mTOR RPTOR |  |
| MAP kinase-activated protein kinase 2 MAPKAPK2 | Hypoxia-inducible factor 1-alpha HIF1A | MAP kinase-activated protein kinase 2 MAPKAPK2 |  |
| Heat shock protein beta-8 HSPB8 | Suppressor of cytokine signaling 3 SOCS3 | Heat shock protein beta-8 HSPB8 |  |
| Signal transducer and activator of transcription 3 STAT3 | Signal transducer and activator of transcription 3 STAT3 | Signal transducer and activator of transcription 3 STAT3 |  |
| Mitogen-activated protein kinase 3 MAPK3 | Mothers against decapentaplegic homolog 2 SMAD2 | Mitogen-activated protein kinase 3 MAPK3 |  |
| CREB-binding protein CREBBP | NF-kappa-B inhibitor alpha NFKBIA | CREB-binding protein CREBBP |  |
| GTP-binding protein Rheb RHEB | CREB-binding protein CREBBP | GTP-binding protein Rheb RHEB |  |
| CCAAT/enhancer-binding protein beta CEBPB | CCAAT/enhancer-binding protein beta CEBPB | CCAAT/enhancer-binding protein beta CEBPB |  |
| Proteasome subunit beta type-1 PSMB1 | Insulin-like growth factor I IGF1 | Proteasome subunit beta type-1 PSMB1 |  |
| Nuclear factor NF-kappa-B p105 subunit NFKB1 | Proteasome subunit beta type-1 PSMB1 | Nuclear factor NF-kappa-B p105 subunit NFKB1 |  |
| Superoxide dismutase [Cu-Zn] SOD1 | Peroxisome proliferator-activated receptor gamma coactivator 1-alpha PPARGC1A | Superoxide dismutase [Cu-Zn] SOD1 |  |
| Sodium-coupled neutral amino acid transporter 9 SLC38A9 | Mitogen-activated protein kinase kinase kinase 7 MAP3K7 | Sodium-coupled neutral amino acid transporter 9 SLC38A9 |  |
| Histone acetyltransferase p300 EP300 | Beclin-1 BECN1 | Histone acetyltransferase p300 EP300 |  |
| GATOR complex protein WDR24 WDR24 | Histone acetyltransferase p300 EP300 | GATOR complex protein WDR24 WDR24 |  |
| Interleukin-6 IL6 | Cullin-1 CUL1 | Interleukin-6 IL6 |  |
|  | TNF receptor-associated factor 6 TRAF6 |  |  |
|  | Interleukin-6 IL6 |  |  |
|  | Proenkephalin-A PENK |  |  |
|  | Phosphatidylinositol 3,4,5-trisphosphate 3-phosphatase and dual-specificity protein phosphatase PTEN PTEN |  |  |
|  | Eukaryotic translation initiation factor 4E-binding protein 1 EIF4EBP1 |  |  |

Table S2: Gene list for pathway analysis of untargeted gene expression

| Gene | Control  admission | Control  Day 7 | FES  admission | FES Day 7 |
| --- | --- | --- | --- | --- |
| PPARγ-C1α | 0.30±1.40 | -1.14±0.57 | -0.10±1.37 | -1.45±1.04 |
| SLC25A20 | 0.29±1.26 | -0.21±0.96 | 0.47±1.45 | -0.03±1.30 |
| UCP-3 | 0.91±1.67 | -0.43±1.51 | 0.91±1.14 | -0.64±1.54 |
| LPIN-1 | 0.33±1.39 | -0.92±1.03 | 0.29±1.33 | -0.71±1.06 |
| SPT-1 | 0.35±1.10 | 0.12±0.83 | 0.23±0.95 | -0.20±1.18 |
| Il-18 | 0.55±2.08 | 1.98±1.33 | -1.30±1.83 | 1.94±1.50 |
| TNFRSF12A | 0.55±2.08 | 1.98±1.33 | -1.30±1.83 | 1.94±1.50 |
| Il-1β | 2.22±1.97 | 0.45±1.98 | 0.92±2.56 | 0.56±2.17 |
| Il-6R | 2.11±1.45 | 0.391±1.26 | 1.27±1.14 | 0.35±1.4 |
| Il-1R1 | 1.76±1.44 | 1.03±0.914 | 1.55±1.26 | 1.08±1.14 |

Table S3: Log_2_ fold change differences in muscle mRNA expression seen in the Primary cohort. FES= Functional Electrical Stimulation; PPARγ-C1α= Peroxisome proliferator-activated receptor gamma coactivator 1-alpha; SLC25a20=Solute Carrier Family 25 Member 20; UCP3= Uncoupling Protein 3; LPIN-1= Lipin-1; SPT-1= SPT1= Serine palmitoyltransferase 1; il-18= Interlukin-18; TNFRSF12A=Tumor necrosis factor receptor superfamily member 12A; il-1β=Interleukin-1 Beta; Il6R= Interleukin 6 Receptor; il-1R1= Interleukin- 1Receptor 1

| Gene | Control | Control | FES  admission | FES Discharge |
| --- | --- | --- | --- | --- |
| UCP-3 | -0.38±1.36 | -3.48±3.15 | -0.86±2.36 | -3.05±1.36 |
| DGKD | -0.57±1.43 | -1.09±0.69 | -0.66±1.82 | -3.59±1.82 |
| Il-18 | -0.27±0.98 | 0.72±1.94 | -0.58±1.47 | 0.93±2.03 |
| MCAD | -0.21±0.98 | -0.75±1.01 | -0.14±0.76 | -0.23±0.67 |
| PYGM | -0.40±1.34 | -2.20±4.15 | -0.33±1.09 | -1.44±0.98 |

Table S4: Log_2_ Fold change differences in muscle mRNA expression seen in the Validation cohort: UCP-3 Uncoupling protein 3, DGKD=Diacylglycerol Kinase Delta; IL-18=Interleukin-18; MCAD=Acyl-CoA Dehydrogenase Medium Chain; PYGM=Glycogen phosphorylase

Figure S3: Targeted fold change differences in skeletal muscle genes regulating intramuscular inflammation between elective hip surgery control patients (CON) and critically ill patients either receiving routine standard of care (SC) or Functional Electrical Stimulation (FES). CXCL3= Chemokine (C-X-C) ligand 3; IL1b=Interleukin 1B; IL1R1=Interleukin 1 receptor 1; IL10RB=Interleukin 10 receptor, beta subunit; IL-18=Interleukin 18; Il-6=Interleukin 6; Il6R=Interleukin 6 receptor; IRAK4=Interleukin-1 receptor-associated kinase 4; MCSF= Macrophage Colony Stimulating Factor; TNFRSF1A=Tumor necrosis factor receptor 1; TNFRSF10B=Tumor necrosis factor receptor superfamily member 10B; TNFRSF12A=Tumor necrosis factor receptor superfamily member 12A; TRAF6=TNF receptor-associated factor 6; TYK2=Tyrosine kinase 2; HIF1A=Hypoxia-inducible factor 1-alpha. Data are expressed as mean ± SEM.
